# Report of Clinical Observations on Continued (Typhus and Typhoid) Fever

**Published:** 1850-10

**Authors:** Austin Flint

**Affiliations:** Prof. of the Principles and Practice of Medicine, and Clinical Medicine, in the University of Buffalo


### BUFFALO MEDICAL JOURNAL

AND

### MONTHLY REVIEW.

**VOL. 6.** OCTOBER, 1850. **NO. 5.**

### ORIGINAL COMMUNICATIONS.

ART. I.—*Report of Clinical Observations on Continued (Typhus and Typhoid) Fever. Based on an Analysis of Fifty-Two Cases.* By AUSTIN FLINT, M. D., Prof. of the Principles and Practice of Medicine, and Clinical Medicine, in the University of Buffalo.

(*Continued from page* 214.)

*Senses and Sensibility.* This division will embrace several sub-divisions. Under the head of *Sensibility,* the occurrence of *pain* in different parts of the body will form the chief topic of inquiry. The morbid conditions of the different senses, but, more particularly, the *eye* and *ear,* will next claim attention.

The part of the body most frequently the seat of painful sensations in Continued Fever, is the *head.* I will examine the cases, therefore, first, with respect to this symptom.

*Cephalalgia.* Of the cases in *private practice,* this symptom is not mentioned in *seven,* and was present in all the remainder, viz., *seven.*

*Three* of the cases in the records of which this symptom is *not* mentioned, came under observation, respectively, on the fifth, sixth and seventh day after the fever was established. In the remaining *four* cases, the fever was observed from its commencement.

In the *seven* cases in which the symptom was present, it was not uniform in degree. It existed in a marked degree in but *two* cases—in the other cases being more or less prominent. It continued from *two* to *four* days, except in *one* case, in which it continued *seven* or *eight* days. In the latter case, it was moderate in degree, and the fever was of a very mild grade of intensity.

In the single case of *Typhus* in this group of cases, which was observed from its commencement, the presence of this symptom is not stated.

In *three* of the *fatal* cases in this group, nothing is mentioned of the presence of this symptom. In the remaining *fatal* case, it existed at the commencement of the disease.

*Hospital Cases.* Of the cases of the *Typhoid* type, cephalalgia is noted as present in *four* only. In *thirteen* cases, nothing is stated relative to this symptom.

In *ten* of these *thirteen* cases, the febrile career had continued for a period not less than *five days*, before coming under observation. In *one* of these cases, the fever had continued but one day before observed; and in *one* case, it was observed from the commencement, i. e., so soon as the patient was compelled to take to his bed. In *two* of these cases, the duration of the disease before coming under observation could not be ascertained.

In none of the *three fatal cases*, in this group, is this symptom stated to have been present. *One* of these cases entered on the *second* day—the *two* others, several days after the commencement of the febrile career.

*In none of the cases of this group in which the symptom was present, did it continue beyond the fourth day.*

Of the cases of *Typhus*, cephalalgia is noted as present in but *two*. In *one* of these cases, it existed for the first day only, the case being observed from the time the patient took to his bed. In the other case, it lasted *two* days, the previous duration of the febrile career not being ascertained. Neither of these cases proved fatal.

In *eight* cases of this group, nothing is noted relative to the presence or absence of this symptom. In *two* cases, it is noted that no pain existed. In *two* of the cases in which nothing is stated relative to the symptom, the fever was observed from the commencement of the career.

Of the cases of *doubtful type*, cephalalgia existed at the early part of the disease, in *two*; of the remaining *six* cases, in *two* it is stated that this symptom was not present, and in *four*, nothing is said relative to it. In *one* of these *six* cases, the fever was observed from the commencement of its career; and in *one*, the patient entered on the second day. In the other *four* cases, the duration of the fever prior to observation was, in one case, *three* days; in one, *five* days; in one, *eight* days; and in one, *six* days. In the single *fatal* case in this group, nothing is stated relative to cephalalgia, the case coming under observation on the *eighth* day.

These results show, *First*—that cephalalgia, in Continued Fever, is not a symptom incident to the disease after the first few days of its continuance. *Second*—that this symptom is present during the first four days, in a certain proportion of cases, but not invariably. The numerical proportion of cases in which it is present, the cases under examination do not afford the means of estimating, so many of them coming under observation after the period in the disease had passed to which it is limited, and the details of the previous history often being imperfectly obtained. *Third*—it is not a symptom which appears to have any connection with the issue of the disease. *Fourth*—in degree, the symptom is variable, occasionally being intense, but, in the majority of cases, not severe or prominent.

It will, of course, be understood that this symptom is now under consideration as it is presented during the febrile career, *after* the access of the disease. It is much more uniformly present during the access than subsequently.*

In general, it is obvious that this symptom possesses small importance, either in a pathological or diagnostic point of view, in Continued Fever.

---

* I wish to state that, in conducting the present analytical examinations, my rule is, to obtain results, and draw deductions, before referring to the works of Louis, and others, in order to ascertain the correspondence or disparity of these results and deductions, with those therein contained. When I express general conclusions with respect to Continued Fever, I always mean those only which the data before me authorize me to draw. If these conclusions differ from those arrived at by other observers, and by means of an analysis of a larger number of cases, then it becomes a question how is the difference to be explained. This question, however, it does not enter into the design of this report to discuss. Nor, as will be observed, do I make it a point to state always how my results and conclusions do compare with those previously obtained by others. I have done so, thus far, in some instances, but not uniformly. My reason for not devoting much space to these topics is, that to give them adequate consideration would swell. too much, the size of the present report. Besides, the report purports to consist only of observations based on an examination of cases that have passed under my own observation. I am led to introduce this note in the present connection, by having noticed, *after* this Section was written, that the facts developed by the researches of Louis, respecting *Cephalalgia*, differ considerably from those I have presented. In his collection of cases, this symptom was found to exist in a much larger proportion of cases, and to have a longer duration. Why this disparity exists, I am not prepared fully to say, but perhaps it may be explained by upposing that in making inquiries relative to this symptom, Louis embraced the period of the *access* of the disease, which, in the plan I have pursued, was excluded. This would also account for the longer continuance of the symptom in the cases observed by him.

It is highly interesting, after having worked out results, to see how they compare with those which have already become the property of science; and one advantage of repeated analytical investigations of the same disease, consists in this comparison, with a view to investigate the causes of differences which may be found to exist.

When unusually prominent, however, and intense, it may occasion perplexity in the mind of the practitioner. This happened in one of the cases occurring in private practice. The pain in the head, in this case, was so severe for the first three days, being accompanied by increased susceptibility to light and sounds, together with considerable general nervous excitability, that I was not without apprehension lest the affection might be encephalitis. The practical importance of not confounding this affection with fever, is too obvious to require remark, and has been already alluded to under the head of *Delirium.* Fortunately, instances are extremely rare in which there exists much ground for hesitation in the discrimination.

The disappearance of Cephalalgia, even when it had been severe, after the lapse of a few days, is an interesting fact in the history of the disease. I would remark that, in observing cases, it appears often as if this fact were owing, not so much to the cessation of the morbid condition upon which the cephalalgia depends, as on the induction of a mental state, rendering the patient unconscious of suffering from the persistence of that condition.

The cases in which, after the career of the fever was established, *pain* in any other part of the body than the head, was experienced, are very few. The histories of the *private cases* contain no account of any other than cephalalgic pain. It is possible that various painful sensations may have existed, but if any had been present, except in a very slight degree, it is probable they would have been recorded.

In the *hospital cases*—(*Typhoid*)—pain in the limbs was the subject of complaint, in *one* case; in back, knees, and other joints, in *one* case; and pain "all over," in *one* case. *Typhus*—pain in the lower extremities, was complained of in *one* case. *Doubtful type*—pain in upper extremities, in *one* case; in the chest and legs, in *one* case; in the back and left hypochondrium, in *one* case; and in the back and limbs, in *one* case.

It may be remarked, that slight, unimportant uneasy sensations, which might have been mentioned, would perhaps not be recorded; so that in the instances above enumerated, the pains were probably somewhat prominent as symptoms.

In a very large proportion of the cases, especially after the first three or four days, the patients made no complaint—frequently declaring they were comfortable, and sometimes that they felt very well. The latter reply was most apt to be returned by those who were the most seriously ill. I have sometimes noticed that a patient who replies to a general question as to his feelings, that he is pretty comfortable, on being inquired of more par-particularly, if he has not some pain somewhere, will apparently deliberate for a time, and afterward refer to some part as the seat of painful sensations. Oftener, after saying he is comfortable, if he be asked, whether he has not pain in the head, abdomen, back, etc., he answers in the affirmative. These observations show that the absence of suffering is due, not alone to the absence of conditions calculated to occasion distress, but to the blunted perceptions incident to the disease.

A blunted perception of morbid sensations was evidenced by various other circumstances; such as indisposition to changes of position, even when the position was such as would ordinarily produce uneasiness; creeping of flies over the face, apparently unnoticed, etc. Circumstances that will be noticed under other Sections, will serve to illustrate the same condition. So that *diminished general sensibility* may be said to belong to the symptomatology of Continued Fever. And it is probably true that, other things being equal, this symptom is, in some measure, proportioned in degree to the gravity of the febrile disease.

I pass now to the *Senses*. And first, the *Eye*.

*Eye*. The observations respecting the eye, relate almost exclusively to the presence or absence of vascular congestion of the conjunctiva. In *two* cases only was diminished mobility of the retina noted. The tone of expression given to the countenance by the eye, has been already considered, under the head of the *general aspect*. Yellowness of the conjunctiva existed in *one* case. In a few cases it is stated, that, while the patient slept or dosed, the eye-ball was partially uncovered; but pains were not taken to record observations with respect to this point. Increased susceptibility to light is not mentioned, except in a single instance—the instance already referred to, in which cephalalgia existed in a severe degree, occasioning the suspicion of encephalitis. As respects *vascular congestion*, of the cases in *private practice*, suffusion, in a marked degree, is noted in *one* case; and in *one* case, the conjunctiva was moderately injected. Both of these were cases of the *Typhoid* type, and in neither did the disease prove fatal. In all the remaining cases, including the case of *Typhus* and the case of *doubtful type*, nothing is stated relative to the appearance of the eye. It is not stated that the eye was suffused or injected, in the instance in which there existed increased susceptibility to light.

Of the *hospital cases—Typhoid*—the eye was suffused in *four* cases. In three of these cases, the degree of suffusion is denoted by qualifying adjectives, as follow:—*slightly*, in *one; moderately*, in *one;* and *somewhat*, in *one.* In *three* cases, it is noted that the eye was *not* suffused; and nothing is recorded in *eleven* cases. It is fair to conclude, that in nearly, if not quite all the cases in the record of which nothing is said on the subject, no congestion existed. Of the four *fatal cases* of this group, in *three* nothing is stated relative to the congested appearance of the eye; and in the remaining case, it was suffused.

Of the cases of *Typhus,* the eye was suffused, or injected, in *six* cases; and in no instance were qualifying adjectives used to denote a light degree of congestion. In *two* cases, nothing is stated relative to the appearance of the eye; and it was *not* suffused, nor injected, in *four* cases. Conjunctival redness was present in the *two* fatal cases in this group.

Of the cases of *doubtful type*, nothing is stated in *five* cases; it is stated that no suffusion existed in *two* cases; slight injection existed in *one* case.

In the use of the terms *suffusion* and *injection,* the former was applied to a watery appearance, with some redness, and the latter to a greater degree of congestion, in which the vascularity was more marked.

A suffused, or injected condition of the eye, thus, is present in a certain proportion of cases of either type of Continued Fever; but the numerical ratio of the cases in which it exists, is not deducible from this analysis; the fact of the absence of this symptom not being noted in a considerable number of cases, although the presumption is, that it was not present in the cases, in the records of which it is not mentioned. The above enumerations, however, show, that it is oftener met with in cases of the *Typhus,* than in the *Typhoid* type; and that it is less in degree in the latter than in the former. The symptom, when present in a marked degree, therefore, has some diagnostic value.

The inquiry arises, whether this symptom be not dependent on the general capillary congestion, which, as has been seen in a preceding Section, is exhibited, in a large proportion of cases, on the face and surface of the body generally. It would be a rational supposition that this was the case; but on comparison of cases, it appears that the two symptoms are by no means uniformly associated. Not only capillary congestion of the skin does not exist in all cases of a congestive appearance of the conjunctiva, but the latter may be absent in cases in which the former is present. Of the *two* cases in *private practice* in which the conjunctiva was reddened, nothing is stated respecting the skin in *one* case, and in the *other* case, congestion of the face co-existed. Of the *Typhoid hospital* cases, in which conjunctival congestion existed, nothing is stated with respect to the skin, in *one* case; capillary congestion of the skin co-existed in *two* cases, and it did not co-exist in *one* case. Of the cases in which conjunctival congestion is recorded absent, capillary congestion of the skin existed in *three* cases.

Of the *Typhus hospital* cases, in which conjunctival congestion existed, nothing is stated respecting capillary congestion of the skin, in *one* case; capillary congestion of the skin co-existed in *five* cases, and was not recorded absent in a single case. Of the cases in which conjunctival congestion was recorded absent, capillary congestion of the skin was present in *four*, i. e., in all the cases.

Hence, it appears, as already stated, that the two symptoms are not constantly associated. This, however, does not prove that they may not both be effects of the same pathological condition—circumstances, which we are not able to appreciate, occasioning the manifestation of either, exclusive of the other.

*Hearing.* Bluntness of the perception of impressions received by the sense of hearing, as a general remark, obtains in Continued Fever, after the first few days of the febrile career, if not from the very commencement. This rule, in so far as the present collection of cases is concerned, would seem to be without an exception. In no case is it noted that, after the third day, there existed a morbid susceptibility to sounds. The only instance in which this is stated to have been the case at any portion of the febrile career, was the case already alluded to, in which unusual cephalalgia and increased sensibility to light existed. Merely dullness of hearing, corresponding with dullness as respects other senses, may be considered as belonging in the category with *diminished general sensibility*, and is probably due to a mental condition.

But in a considerable proportion of cases, the sense of hearing is affected specially, and in a degree disproportionate to the state of the mental perceptions.

*Deafness*, more or less, is noted in the records of *six* of the *fourteen* cases in *private practice*. Of the *hospital cases*, this symptom is stated to have been present in *five* of the *eighteen* cases constituting the *Typhoid* group; in *five* of the *twelve* cases of *Typhus;* and in *two* of the *eight* cases of *doubtful type*. Generally, in the cases in which it is not stated that the symptom was present, nothing is said on the subject. It is fair to presume that in all, or nearly all of the cases in which the symptom was discoverable, or sufficiently marked to be observed, it was embraced in the records. But it was not always easy to determine as to its existence. In cases of marked somnolency, or stolidity, and when persistent active delirium existed, it was sometimes difficult to appreciate the existence of deafness.

Hence, I do not suppose that the above enumerations possess much value in endeavoring to ascertain the precise ratio of frequency in the occurrence of this symptom. But here, as in many other instances, arithmetical accuracy is not of great practical importance. It is sufficient to form a rough estimation of the probabilities of its occurrence.

Nothing appears, on an examination of the cases, to denote a connection between this symptom and other events of the febrile career. It was present in cases of a mild as well as severe grade of intensity. It was observed in cases of both types, but oftener in Typhus. It was associated sometimes with somnolency and delirium, but was also noted in cases in which the latter symptoms were slight or absent. Of the hospital cases, of both types, which proved fatal, it was noted as present in *two*. In *one* case it was noted absent; and in the histories of the *three* other cases, nothing is said relative to its presence or absence.

It was observed that, when deafness existed, it frequently, if not generally, persisted through the febrile career; and, in some instances, continued for some time after convalescence was established.

It was by no means uniform in degree; sometimes occasioning only some bluntness of hearing—but in some cases rendering it necessary to speak quite loudly in order to be heard.

In some cases patients complained of the difficulty they experienced in hearing, and in other cases it did not appear to excite their notice.

No other facts relating to the sense of hearing were recorded, except that in *two* instances, in connection with deafness, the patients complained of a noise or buzzing in the ears.

As regards the senses of *smell* and *taste*, pains were not taken to record, or to make observations.

*Involuntary Muscular Contractions.* Involuntary muscular contractions were noted in so small a number of instances, that it will not be tedious to notice them and their peculiarities, individually.

Of the cases in *private practice*, involuntary muscular contractions are stated to have existed in *three*. In *one* of these, they consisted in *subsultus tendinum*, and visible twitching of the hands during sleep. The patient, in this instance, recovered. The disease, in its general features, did not present great gravity.

In *one* of the other instances, there existed marked tremulousness of the hands and the tongue, at the commencement of the disease. So prominent was this symptom that, for the first day, the affection was supposed to be delirium tremens—the character and the history of the patient being unknown. This case ended fatally on the *fourth* day, and was of the *Typhus* type.

In the *remaining* case, there existed tremulousness of the hands and muscles of the face, when the patient was first seen on the *fifth* day. The case ended fatally on the *ninth* day. This case was of the *Typhoid* type.

Of the *hospital cases*—*Typhoid*—in *one* case, in which recovery took place, some tremulousness of the hands was observed, on one day. The case was one of severity. In another case, there existed *subsultus* and marked tremulousness of the hands, together with inability, on one occasion, to protrude the tongue. This case ended fatally on the *tenth* day.

Of the cases of *Typhus*, *subsultus* was noted in but *one* case. This was a case of considerable, but not very great severity, ending in convalescence on the seventeenth day after entrance—the patient entering twelve days after illness commenced.

In the two fatal cases of this type, in *one* the power of articulation was lost toward the close of life—the patient not being unconscious. In the other case, much difficulty was experienced in protruding the tongue—repeated efforts being required. Coma became developed in the latter case.

Of the cases of *doubtful type*, tremulousness of the muscles, toward the close of the disease, was noted in the case which proved fatal. Nothing under this head appears in the records of the other cases in this group.

The instances in which this symptom is recorded as present, are fewer than I should have estimated before the analysis was made. A symptom so obvious and important would not be likely to be overlooked or omitted in the history. It is, however, possible that it may have been present, in the form of *subsultus*, and in a minor degree, in some cases in which it was not manifested at the diurnal examinations and records, and, hence, have been omitted.

It will be observed that, in most of the cases in which involuntary muscular contractions were noted, the disease ended fatally. In view of this fact, taking also into consideration the fact that the symptom is one of rare occurrence, its presence is of bad omen, as respects the prognosis.

*Carphologia.* This symptom properly belongs among the aberrations of the sense of vision, consisting in voluntary motions directed toward illusory objects in the air, or on the bed-clothes. It was noted in *three* cases. In one case, of the *Typhus* type, it was observed only on one day. The disease in this instance was severe, but did not prove fatal.

In *one* case, the type was *Typhoid*, and the disease ended fatally. The case was characterized by vigilance, and very active delirium, up to a few hours before death. It was a prominent symptom in this case, in the latter stage of the disease. In the remaining case, the type of the fever is classed as *doubtful.* The patient entered the hospital on the third day after the attack; but the fever had supervened on an illness of three weeks' duration, (the character of which was not ascertained,) from which he had but recently recovered sufficiently to return to labor. Convalescence was pronounced in this case on the sixth day after his entrance.

*Pulling up of the bed-clothes*, I do not find noted except in one case. It may possibly have existed, and, not being apparent at the time of the daily examination, escaped being recorded. In the case in which it was present, it was associated with Carphologia. This case was the last of the three cases mentioned above.

*Prostration.* By *prostration,* I mean exhaustion, or reduction of the force inherent in the voluntary muscular system. Under this head I shall make a few general remarks, based on an examination of the cases, without any enumerations. The latter cannot, in this instance, be made of much avail, inasmuch as we have no means of measuring, with precision, the deterioration of voluntary muscular power. We can only form estimates which are but rough approximations to correctness, and these must be expressed by terms somewhat indefinite. For examples, we may say, that the prostration is moderate, slight, considerable, great, very great, etc. These expressions, obviously, have relative significations, which, taken in the connection in which they are used, may be sufficiently explicit for practical purposes, but they lack that definiteness which is desirable in data for for numerical results.

In a certain sense, fever invariably induces a considerable degree of muscular prostration. The event selected to indicate the commencement of the febrile career, is evidence of this fact. Patients are compelled to take to the bed. They are unable to maintain the erect posture, or to continue, except for a short time, efforts of locomotion. In saying of a particular fever patient, he is greatly prostrated—it is not to be understood that the powers of a healthy, vigorous man are taken as the standard of comparison; but that his muscular strength is reduced considerably below an average of the degree of diminution incident to the febrile state. Judged by this rule, in the majority of the cases which form the subjects of the present analysis, the prostration was not great. The patients generally were able to raise themselves in bed, to change their position by their own efforts, etc. The loss of muscular power was not so great as in many protracted chronic diseases in which the patient is obliged to keep the bed.

In a considerable proportion of cases, patients were able, with the assistance of an attendant, to get out of bed, in order to pass their evacuations, for change of bed-linen, etc. Sometimes they were able to remain sitting for some time; and, when this was not the case, it frequently appeared to be owing rather to *giddiness*, than to a deficiency of muscular force.

The loss of muscular power was often more apparent than real. There was ability to exert considerable voluntary effort, but a mental indisposition thereto. The nervous, rather than muscular force, was at fault. The streng h was more oppressed than reduced. Owing to mental dullness, it was difficult, sometimes, to rouse the patient to a trial of voluntary effort; and in cases in which marked somnolency, or a tendency to coma existed, it was not easy, on this account, to form an estimate of the amount of muscular ability retained by the patient.

In cases in which the fever was of a mild grade of intensity, there existed moderate or even slight prostration, patients being able to assist themselves with very little aid from others. Extreme prostration always occurred in connection with other symptoms denoting unusual gravity of disease. The converse of this, however, was not true. In some of the cases attended with most danger, and some of those ending fatally, the muscular strength was retained in a surprising degree. In two fatal cases of the *Typhoid* type, characterized by active, persistent delirium, the muscular efforts were almost constant, and quite strong up to a few hours before death. One of these cases terminated on the ninth day, and the other on the third day after coming under observation. The mode of dying, in each, was by *asthenia*, or, perhaps, more properly, *necrœmia*—the system of involuntary muscles exhibiting reduction of force to a degree incompatible with life—the voluntary muscles remaining active. This is a curious fact.

It follows from the above observations, that while an unusual degree of prostration is an unfavorable sign in Continued Fever, the preservation of considerable muscular force is not to be considered as, correlatively, a favorable sign.

In several of the cases, I have observed a fact which has an important practical bearing upon the treatment of fever, viz., an improvement in strength during the febrile career. In the histories of the cases, it is occasionally noted, that the prostration at first apparent, diminished while the disease was yet in progress. In these cases, the patients had not received proper care in the way of nursing, or medical attendance; and in some instances, according to the views of the writer, the management had been not only defective, but injudicious. As respects the average degree of muscular force presented in a series of fever cases, much, I am persuaded, will depend on the therapeutical system pursued. This, however, is a topic which will more appropriately come up under the head of the Treatment of Continued Fever.

On consulting the Treatise on Fever by Louis, the reader will find that the results under the head of *Strength*, appear to be at variance with the foregoing. If I may be permitted a criticism upon the observations of that distinguished observer, under this head, I should say, that he does not seem to distinguish between the absolute loss of muscular power and the reluctance to exert it. If the reader will carefully peruse his remarks on this topic, I think he will discover sufficient evidence of the correctness of this criticism.

Louis gives an account of some cases in which the muscular strength was retained sufficiently for the patient to keep about several days after the fever was established. Agreeably to the rule I have adopted, these patients would not be considered to be laboring under established fever prior to the time they took to the bed. As I have before stated, this rule is somewhat arbitrary, and does not mark the commencement of the disease, with exactness, in all cases. Nevertheless, it seems to me to answer better as a point of departure for the origin of the disease, than any other event, or collection of events, that can be selected.

### SECTION FIFTH.

*Symptoms referable to the Digestive System. Appetite. Thirst. Tongue. Sordes. Parotitis. Nausea, and Vomiting. Alvine Dejections. Tympanites. Tenderness of Abdomen. Gurgling.*

Morbid conditions of the digestive system are involved in important questions relating to the diagnosis, pathology and therapeutics of Continued Fever. That portion of the symptomatology of the disease, therefore, which is embraced in this Section, opens a field of inquiry from which interesting, if not useful, results may be hoped for. I will take up the several sub-divisions of the Section in the order in which they are enumerated in the caption.

*Appetite.* The importance of noting negative as well as positive facts, here, as in several other instances, being overlooked in recording the histories of the cases under analysis, the data are too defective for any statistical results. In a large number of the cases nothing is stated relative to the appetite. It cannot be doubted, however, that *anorexia* exists in the vast majority of cases of Continued Fever; and it is to be presumed, that this symptom was present in most of the cases in which the records are silent on the subject.

In a few cases it is noted, that appetite existed—the patients declaring that they took the food given to them with some relish. In connection with this fact should be stated, what will be noticed hereafter under the head of *Treatment*, that the majority of the patients were allowed milk porridge and essence of beef, for diet, during the greater part of the febrile career. In *two hospital* cases, of the *Typhoid* type, the patients said, that the diet was agreeable; in *one* case of *Typhus*, the same is noted; and in another case of the same type, it is stated that the patient had some appetite. In all the other cases, both in private and hospital practice, either anorexia existed, or nothing appears in the histories relative to this subject. The reader may have some curiosity to know if there were any characters distinguishing the *three* cases in which food was relished, and the single case in which it was desired. Of the two *Typhoid* cases, *one* proved fatal. The patient, in this case, was passively delirious during the early part of the disease, but the mind subsequently became clear. He was greatly prostrated. Evacuations were passed in bed, not unconsciously, but from inability to restrain them. Death occurred, twenty-six days after the attack, by exhaustion, (*necrœmia.*) In this case, anorexia existed part of the time. In the other case, the disease was of a mild grade; the patient being convalescent eight days after entering hospital, and quite well on the nineteenth day. In the two *Typhus* cases, both patients recovered. The disease, in both, was of medium severity. In both, pulmonary symptoms were prominent. In one, it is stated that food was relished through the febrile career ; in the other, appetite was stated to exist only on one day—the second day after admission. No circumstances thus appear which can afford any explanation of the deviation from the general rule, as respects the appetite, in these four cases.

I would remark, that I do not feel confident that the four cases just mentioned, were the only instances in which food was relished, not being satisfied that proper pains were taken to make inquiries and notes relative to this point, during the examination of patients.

*Thirst.* Of this symptom, as of that just noticed, owing to want of care to record negative facts, I cannot submit any statistics as respects the frequency of its occurrence, its duration, etc. In a large proportion of the cases, the records contain no reference to thirst. I will not presume that it did not exist in any of these cases, but I am confident it was not a prominent symptom in any of them during the time they were under observation, after the fever became established. In *three* of the cases in *private* practice; in *three* of the *hospital* cases of *Typhoid*, in *two* of *Typhus*, and in *one* of *doubtful* type, *thirst* is mentioned as a symptom continuing, more or less, through the febrile career. In all these cases, save two, the thirst was moderate in degree. It is also worthy of remark, that in all, save the two last referred to, the disease was of a mild grade of severity; and in *four* of the cases, very mild. In the *two* cases in which thirst was more than moderate, the disease proved fatal. One of these cases was among the number in which food was relished; and in this case, the thirst was considerable. In another instance, thirst was associated with the relish for food.

In addition to the *nine* cases in which, as just stated, *thirst* existed through the career of the fever, it is noted to have been present in the early part of the disease in several instances, viz., in *four* cases of Typhoid, *three* of *Typhus*, and two of *doubtful type*. It will be recollected that in a large proportion of the *hospital* cases, the patients did not come under observation at the earliest stage of the disease. It is altogether probable that, in several of these cases, this symptom had existed and ceased prior to the period when the records commenced.

The cessation of the symptom is readily explained by attributing it to the bluntness of perception which usually characterizes the disease after the first few days. The morbid conditions which had occasioned the sensation of thirst, probably continue; but the faculty of perceiving these conditions, or of suffering in consequence of them, is no longer acute. This idea is confirmed by a fact above noted, viz., that in most of the cases in which thirst continues through the career, the disease is of a mild grade—less obtuseness of sensibility belongs to its progress. It is also confirmed by another fact which I have very generally observed, but which is not noted in the histories, viz., that although the sensation of thirst may not create sufficient uneasiness to lead the patient to ask for drink, nevertheless, when it is presented, he almost always takes it with readiness, and frequently with avidity, showing the existence of the symptom, but, as it were, latent, owing to the mental apathy peculiar to this disease.

*Tongue.* The morbid appearances of the *tongue* are observed with attention in all diseases; but in few, if any, are they generally regarded as possessing greater interest and importance than in febrile affections. Some practitioners attach a significancy and value to particular conditions of this organ, which close investigation probably will not warrant. Certain appearances are supposed to stand in a fixed relation to certain pathological conditions of other parts, and of the system at large; or, they are thought to foreshadow the issue of the disease, or to indicate particular methods of treatment. These notions, which it might be presumed involve both truth and error, are partly empirical and partly speculative—that is to say, in some instances, theoretical views have doubtless had more or less to do in originating them; but it is also frequently claimed that they are corroborated by experience. In either case, the analytical investigation of a large number of recorded observations, is the proper tribunal for adjudication. By ascertaining precisely the position which every appearance occupies in the natural history of the disease, its importance as a symptom or sign is determined.

There are two points of view in which the appearances of the tongue in Continued Fever are to be studied. *First*, the different appearances which are found in cases of the disease, considered collectively, their relative frequency of occurrence, their relations with other symptoms, etc. *Second*, the different appearances which are successively presented during the progress of the disease, in individual cases. In both points of view, the appearances are various.

Of the *fifty-two* cases which are the subjects of the present analysis, the histories contain an account of the appearances which the tongue presented during the febrile career, in all but *two* cases. In not one of these cases did the tongue retain a perfectly normal aspect through the career of the disease. The nearest approximation to this was in one of the cases in private practice—(*Typhoid*)—in which the disease was of a very mild grade, the patient being convalescent on the *fifth* day. In this case the tongue remained perfectly natural in appearance until a couple of days before convalescence, when the superior surface became slightly opaque, hardly enough so to be called furred, and not more than is habitually observed in many persons who call themselves well. In all the other cases morbid appearances, more or less considerable, were presented. The different appearances presented in the different cases, involve varying degrees of dryness of the surface of the organ, together with resistance to the touch; coatings varying in thickness; varieties in *color;* smoothness, fissures, etc ; aberrations of muscular contractility, or an inability to protrude it, facility of performing this act, etc. I will take up the more important of these appearances separately.

*Dryness of the Surface.* More or less dryness of the tongue was present in a large proportion of the cases, viz., in *six* of the *fourteen* cases in *pri-*

*vate* practice; in *fifteen* of the *eighteen hospital* cases of *Typhoid, ten* of the *twelve* cases of *Typhus,* and *five* of the *eight* cases of *doubtful* type; making, in all, *thirty-six* of the *fifty* cases in which the appearances of the tongue were noted.

The dryness differed in degree in the different cases; in some being slight, in other cases extreme, and in others, moderate. In several cases, the dryness was accompanied with *hardness,* that is, the surface presented an appearance as if desiccated or baked, offering firm resistance to the touch. This was observed in *two* of the cases in *private* practice; in *three* of the *hospital* cases of *Typhoid,* in *four* of the cases of *Typhus,* and in *one* of the cases of *doubtful type.*

The dryness, in some cases, extended over the whole superior surface, and, in other cases, was limited to the centre—a moist margin, of variable width, existing on either side. The latter was observed in *one* of the cases in *private* practice; in *eight* of the *hospital* cases of *Typhoid,* in *four* of the cases of *Typhus,* and in *two* of the cases of *doubtful type.*

The dryness was in no case observed at the commencement of the febrile career. In all the cases that came under observation at the commencement, the dryness did not appear until several days had elapsed; and in every instance in which dryness was present at the time the case was first observed, the disease had existed for several days. This condition, therefore, although so frequently present in Continued Fever, does not belong to the early stage.

What does this symptom denote? in other words, what are the causes which produce it? Diminution of the secretions from the salivary glands and mucous follicles is, doubtless, a cause; but it is not, probably, the sole cause. It proceeds, in part, from the condition of the mind incident to Continued Fever. Owing to the apathy as respects painful sensations, the patient appears to experience little or no discomfort from the dryness and hardness of the tongue. He, therefore, does not move the organ sufficiently to diffuse the scanty salivary secretion over its surface. The tongue, moreover, participates in the inertia of the muscular system. It remains motionless. The somnolency which obtains in the majority of cases, contributes to this symptom. The patient lying most of the time somnolent, respiring through the mouth, (the nasal passages frequently being more or less obstructed,) the surface of the tongue is desiccated by the current of air passing over it.

The correctness of the above explanation is proved by the following considerations:—Patients very seldom make any complaint of the condition of the tongue, however dry and hard it may be. The dryness and hardness do not come on during the early part of the febrile career; nor, (as has been seen in the preceding Section,) do somnolency, muscular inertia, and mental apathy. The former and the latter occur concurrently, at the same period in the febrile career; and, as a general remark, it will probably be found that they disappear together. Finally, the degree of dryness and hardness will be found to bear a certain degree of correspondence with the ataxic symptoms just mentioned; nor are the former present, as a general rule, in cases in which the latter are absent. In evidence of the statements last made, I have examined,—*first*, the symptoms referable to the nervous system in all the cases in which dryness of the tongue was *not* present, and the results are as follows:—of the *eight* cases in *private practice*, in which dryness of the tongue was not noted, in *one*, the tongue could not be inspected, except at first, owing to parotitis; in one, the patient died on the third day; and in one, the records contain nothing on the subject. Of the remaining *five* cases, somnolency, muscular inertia, and mental apathy, were not marked in *one* case, and were almost absent in each of the other cases, all of which were extremely mild as respects the grade of severity of the disease. Of the *hospital* cases, in the two instances of *Typhoid*, in which dryness of the tongue was *not* noted, active delirium, followed by coma, existed in *one* case, the patient dying on the sixth day; in the other case, ataxic symptoms were absent. In the *two* cases of *Typhus*, in which dryness of the tongue was *not* noted, in *one* case, the tongue could not be inspected, owing to parotitis, and in the other case there existed hilarity and playfulness, without either somnolency, muscular inertia, and mental apathy. Of the *three* cases of *doubtful type* in which dryness of the tongue was *not* present, in each case the nervo-muscular symptoms just mentioned were absent. *Second*, I have examined the symptoms referable to the nervous system in the cases in which dryness and hardness of the tongue existed, and find in all but *two*, (in which the records are defective,) evidences of the presence of somnolency, muscular inertia, and mental apathy—in other words, of ataxic symptoms. As the histories do not state the degree of these symptoms, I am unable to ascertain whether, in that respect, they corresponded with the degree of dryness which the tongue presented.

In conclusion, then, we are warranted in saying that, although dryness and hardness of the surface of the tongue denote deficiency of the secreted fluids poured into the mouth, they are to a considerable extent dependent on the condition of the mind and muscular system, and may be considered, to some extent, as measuring the disorder of the latter.

*Coating.* In *forty-seven* of the *fifty* cases in which the appearances of the tongue were noted, it was more or less *coated* during the febrile career. Of the remaining *three* cases, (all of which were of the *Typhoid* type,) in *one* there does not appear to have been any coating; in *one,* there was none for the first two or three days after the case came under observation; but the tongue could not be inspected afterward, owing to parotitis, affecting both sides; and in *one,* there was merely a slight opacity, hardly sufficient to be called a coating. Under the head of *Coating,* I mean to include all instances in which a morbid secretion, or exudation, was deposited on the superior surface of the tongue. It is common to distinguish a *furred* or *frosted* tongue, from one which is *coated,* the latter being applied when the morbid deposit is of an appreciable thickness. For the sake of convenience, however, I have used the term *Coating* in a sense broad enough to embrace the former as well as the latter. The coating varied much in the different cases as respects thickness. All of the histories do not contain precise information on this point, but, in the majority of instances, it is stated either that the tongue was *thickly coated,* or *thinly coated,* or merely *furred.* The enumerations with regard to these three modifications of coating are as follows:—Of the *eleven* cases in private practice, in which coating is noted, it is simply stated, that the tongue was *coated* in *three;* it was *thickly coated* in *four;* it was *thinly coated* in *two,* and *furred* in *two.* Of the *eighteen hospital* cases of *Typhoid,* it was *coated* in *four; thickly coated* in *two; thinly coated* in *six; furred* in *five,* and no coating existed in *one* case. Of the *twelve* cases of *Typhus,* it was *coated* in *five ; thickly coated* in *three; thinly coated* in *two; furred* in *one* case, and the organ could not be inspected, owing to the existence of *parotitis,* in *one* case.

Of the *eight* cases of *doubtful type,* it was *coated* in *four; thinly coated* in *two; thickly coated* in *one* case, and *furred* in *one* case. The cases of the different types of Continued Fever presenting respectively these differences in thickness of the coating, are not uniform in number, but the discrepancy is not such as to warrant the conclusion that either modification is more likely to occur in the *Typhus,* than in the *Typhoid* type. Not less disparity would, very likely, be found on comparing different collections of cases of the same type, than in the comparison, in this particular, which these cases afford of the two types. We should not expect to discover in the varieties of this symptom any traits distinguishing one type of the fever from the other, inasmuch as these varieties are based on differences in degree merely, not in kind, and, moreover, are common enough in other, and, indeed, in almost all affections.

There seems to be no room for the inquiry whether the coating has any connection with any other particular symptoms, or group of symptoms, belonging to the natural history of Continued Fever. Occurring, as the symptom does, in almost every case of Continued Fever, it must be dependent on some one or more of those morbid changes which constitute the elements of the febrile state. Farther than this, in the present state of knowledge, we are unable to carry the explanation of this, as well as various other symptoms appertaining to the disease under consideration.

The only remaining points of inquiry which occur to me under this head are, *first*, to examine the histories of the few cases in which the tongue was not coated, in order to see in what respect they differed from the others; and, *second*, to compare some of the cases in which the tongue was *thickly coated*, with some of those in which the coating was *thin*, in order to see if the degree of coating affords, to any extent, a criterion of the severity of the disease.

As already stated, in but *three* cases is the absence of coating noted, and in *one* of these cases the tongue could not be inspected after the first two or three days, owing to parotitis. The patient, in this case, at the time she was attacked with fever, was suffering from *stomatitis materna*, and the tongue presented a characteristic reddened, excoriated appearance. One of the remaining cases was the case in which the tongue preserved its normal appearance save a very slight opacity. This case was distinguished for its mildness, and short duration. In the other case, the tongue was reddened, glazed, and somewhat fissured. The patient, in this case, had been ill eleven days before entering the hospital. It is highly probable that there had existed coating of the tongue, which had exfoliated prior to his entrance. The case was one of medium severity, and was not distinguished by any peculiar features. Convalescence was pronounced on the fourteenth day after his entrance. It will be observed that the circumstances appertaining to each of these cases were such that the latter are hardly to be considered exceptions to the general rule, as regards the uniform existence of more or less coating of the tongue in the present collection of cases of Continued Fever.

With regard to the second point of inquiry, I have examined and compared cases sufficiently to ascertain that the thickness of the coating cannot be considered as denoting the gravity of the disease, or the danger of the patient. Whether, in the larger proportion of severe or fatal cases, the tongue may be thickly, or thinly coated, I am not prepared to say—the present collection of cases is too small to afford statistical results adequate to the settlement of this question. But that, if any such law exists, the exceptions are sufficiently numerous and striking to render its application to individual cases nugatory, my cases afford sufficient evidence. Of *two* of the fatal cases of *Typhoid*, the tongue was merely *furred* in *one*, and, in the other, at first furred, and subsequently thinly coated. In *one* of the fatal cases of *Typhus*, it was thinly coated. On the other hand, in several cases in which the tongue was thickly coated, the disease was mild. In the fatal case of *Typhus* occurring in private practice, in which death occurred on the morning of the fourth day, the tongue presented a normal aspect, except that it was tremulous, for the two first days, and became thickly coated on the third day.

It appears pretty obvious that very little importance is due to the degree of coating on the tongue in any of the practical relations, or bearings, in which the symptoms of disease are to be considered.*

The coating of the tongue, in the majority of cases, appeared early in the disease, preceding *dryness*. The tongue, however, was not thickly coated at first, in several cases in which it became so at a subsequent period. It was frequently at first simply furred, and in the progress of the disease became thickly coated. As a general rule, the coating became thinner toward convalescence, and gradually disappeared—leaving the appearance of the organ healthy. In a few cases, however, the tongue continued somewhat coated after convalescence was established.

The color of the coating was generally white, or dirty white, sometimes yellowish. In *seven* cases it became of a dark color. *Four* of these were cases of *Typhoid; two* in *private practice*, and *two* in *hospital; two* were cases of *Typhus*, and *one* was a case of *doubtful* type. In *one* of these cases the disease proved fatal, but in the other cases the disease was either mild, or of a medium grade of intensity; so that there is very little, if any, foundation for the idea that the dark, or black color of the coating, in cases

---

* The foregoing results relating to *dryness of the surface of the tongue*, and *coating*, appear to present a striking disparity on comparison with those developed by the researches of Louis. He found in a considerable number of cases, the tongue natural, or nearly so. This was true of grave and fatal cases as well as of those of a mild grade. The appearances which the tongue presents are so obvious, and offer so little room for error of observation, that, with every disposition to distrust my own ability as an observer, I cannot admit the supposition of incorrectness, in the histories I have collected.

In the cases analyzed by Dr. Jackson, the tongue appears to have been more uniformly altered than in the cases analyzed by Louis. Dr. Jackson says, "with few exceptions the tongue was noted as coated, or furred."

of fever, denotes extreme danger. This idea is entertained by some, who regard the coating as representing a perverted state of the fluids, and the dark color as denoting putrescency.

*Exfoliation of the coating* during the career of the disease, occurred in *three* cases—*one* case being of the *Typhoid*, *one* of the *Typhus*, and *one* of *doubtful* type. In the first of these three cases the tongue became subsequently dry and hard, and another coating succeeded. In the *two* other cases the tongue, directly after the exfoliation, was moist, and of natural color, and the occurrence of a second coating is not mentioned. In each of these three cases the disease was mild.

A *scabby* appearance was noticed in a few cases, viz., *two* cases of *Typhoid*, and *two* of *doubtful* type. This appearance was owing apparently to dryness and coating co-existing, the morbid deposit on the tongue becoming cracked and subdivided into small portions, which were partially detached.

In *two* cases, *one* of *Typhus*, and *one* of *doubtful* type, the coating was in discrete patches; but in all the other cases it was either diffused over the whole superior surface, or extended across the base, and, more or less, toward the tip, or covered a margin on each side of the whole length of the organ.

The pathological explanation of coating does not fall within the scope of this report, but the reflection arises, how little we know of the causes of this symptom in any, and all of its varieties—in other words, of the morbid changes of which the symptom is the immediate effect. Its mysterious character is perhaps less appreciated on account of its being so common. But that a morbid product should be deposited on the surface of the tongue, without any other appreciable evidences of disease of that organ; that the presence of this product should so often denote the continuance of disease, and its disappearance signalize the occurrence of convalescence, is certainly a curious fact, and one which, in the present state of knowledge, cannot be accounted for. All that is known of this symptom, as a symptom, or sign, has been obtained by observing its association with other events, and in all diseases, as well as in Continued Fever, its special significancy is quite limited.

*Redness of the tongue.* The superior surface of the tongue was reddened in *seven* cases of the *Typhoid*, and in *one* case of *doubtful* type. This appearance was not observed in any of the cases of *Typhus*. In *one* of the cases of *Typhoid* the redness was associated with *stomatitis materna*, or the sore mouth of nursing women, under which the patient was laboring when attacked with fever. In *two* of the remaining cases, the disease proved fatal. In one of the fatal cases, the disease was characterized by active delirium. Vomiting was not present, nor diarrhœa. The only gastric symptom was thirst, which was, at first, considerable. At the autopsy, in this case, there were found punctated redness and softening of the mucus membrane, together with several ulcerated patches. The papillæ of the tongue were unusually prominent in this case. In the other fatal case, vomiting, and other gastric symptoms, are not mentioned, and diarrhœa did not exist. Moderate tympanites and abdominal tenderness were present. At the autopsy the stomach was not examined. In the *four* remaining cases the disease was not severe, save in one case. Symptoms indicative of disorder of the digestive tube, in these cases, respectively, were as follows:—In *one*, moderate diarrhœa—no vomiting. Convalesced on the tenth day after entrance, and left the hospital on the sixteenth day. The redness of the tongue, in this case, continued after convalescence was established. In *one*, neither vomiting, nor other symptoms of unusual gastric disorder were present, and diarrhœa did not exist. In *one*,—the same as in the last case. This case was tolerably severe. In *one*, no vomiting or other gastric symptoms, and diarrhœa was not prominent. In the case among those of *doubtful* type, no gastric symptoms were present, but moderate thirst, at first. Diarrhœa did not exist, and distension and tenderness of the abdomen were absent.

The fact that in no case of *Typhus* was redness of the tongue present, is perhaps worthy of note.

I have given this summary of the other symptoms referable to the digestive system in the cases in which a reddened tongue was observed, because the idea still prevails, to some extent, with practitioners, that this appearance denotes either inflammation, or notable irritation of the mucus membrane, lining the digestive canal, and more particularly the stomach. In so far as symptoms are concerned, the above instances afford no evidence in support of this hypothesis. The fatal case in which the stomach was examined, it is true, was characterized by gastric disease which was not indicated by gastric symptoms during life. The researches of Louis, however, have established, conclusively, that in fatal cases of Continued Fever there does not exist any constant connection between the appearance of the tongue, and the existence of lesions of the stomach.

*Tremulousness of the tongue.* Tremulousness was observed in *seven* cases, viz., in *three* of *Typhoid*, and in *four* of *Typhus*. *Three* of these were fatal cases. In the other instances in which this appearance was noted, the disease was not of very great severity. Nor was this appearance associated with singultus, tremor of other muscles, or marked disorder of the nervous system, in any case excluding the fatal cases. In *one* of the cases the tongue continued somewhat tremulous during convalescence, and even at the time the patient left the hospital, six days after convalescence was established, remaining, also, somewhat reddened. A tremulous state of the tongue, then, cannot be considered as representing a condition of the muscular system in general, nor as an indication of the degree to which the nervous system is affected. And although, as it would seem, it is apt to occur in cases which prove fatal, its presence need not affect very materially the prognosis, since it also occurs in cases in which the disease is of a medium grade of intensity.

*Difficulty of protruding the tongue.* This existed, to a greater or less extent, in *six* cases, viz, in *two* of *Typhoid*, *three* of *Typhus*, and in *one* case of *doubtful* type. In two of the *Typhoid* cases the difficulty was not great. The patient only did not readily succeed in the effort to protrude the tongue. In the other *Typhoid* case the patient was wholly unable to accomplish the protrusion, but this inability existed during one day only. This was the day before the decease; and on the day of the decease he succeeded in protruding the tongue without great difficulty. In this case, also, there was loss of the power of articulation for the last two days of life. The patient could be roused to make an effort to speak, moving his lips, but uttering no articulate voice. In the *two Typhus* cases the difficulty of protruding the tongue was great. One of these cases was characterized by apoplectic coma, and there existed great difficulty in moving the upper extremities, as well as the tongue. In the other case the patient exhibited unusual somnolency, which eventuated in coma. This case ended fatally. In the case of *doubtful* type, there existed inability to protrude the tongue on the first day of entrance, unusual feebleness of intelligence co-existing. The patient could not be made to reply to questions. The ability to protrude the tongue was recovered on the following day, and, at the same time, there was improvement in the state of the intellect. In all the cases, save one, the difficulty in protruding the tongue, corresponded, in degree, with the diminished power of exerting acts of the will to produce voluntary motions; and in the excepted case, the difficulty was inconsiderable in degree. It would seem, therefore, that this symptom, in so far as it may possess any special significance, is more a criterion of the condition of the mind, than of the muscular force.

A remark suggests itself in this connection which is not limited in its application to cases of Continued Fever. It is, that patients whose intelligence is so far compromised that they cannot be made to reply to questions, may often be induced to protrude the tongue, or to make an effort to do so. The mental apprehension seems sometimes not to extend beyond the request to perform this single act. I know of no way of accounting for this curious fact except that, as the inspection of the tongue always enters into the daily examination of cases of disease, it becomes so associated with the visit of the physician, that, if a patient retains mind enough to recognize his medical attendant, he almost instinctively apprehends the request to protrude the tongue. It requires, from the association just mentioned, less mental effort than to understand and comply with other requests.

More or less hesitancy in protruding the tongue was frequently observed, although pains were not taken to note observations concerning this point in the histories. It appeared, often, as if the patients took time for deliberation before exerting the act of volition, and the protrusion was made very slowly, as if the organ were not fully under the control of the will. This, doubtless, proceeds from the same mental condition which, when greater in degree, leads to difficulty in performing the act, or entire inability to accomplish it. Instances were also noticed in which, when the tongue was protruded, the patient seemed to forget to withdraw it, and it was necessary to request him to do so.

A few appearances were noted in addition to those already considered, which I will mention, in order to embrace *all* that the histories contain with respect to the tongue. The tongue itself became *fissured*, or *cracked*, in *four* cases, all of the *Typhoid* type. In *one* of these cases, the fissures did not heal for several days after convalescence was established. In all, the *fissures*, or *cracks*, occurred while the surface of the organ was dry and hard. The superior surface was noticed to be remarkably *smooth* in several (*five*) cases; and in *three* of these cases it presented a *glazed* appearance. The *smoothness* was apparently owing to dryness, the tongue being clean, and the *glazed* appearance to dryness with a thin stratum of coating.

Finally, in one case, indentations of the teeth on the sides of the tongue were noted

Variations in the volume and form of the tongue, if they existed, were not recorded.

The different appearances of the tongue found in the cases collectively, having thus been considered, it remains to direct attention to the second point of view under which this class of symptoms are to be studied, viz., the different appearances which are successively presented in individual cases. This view of the subject has been, in a measure, anticipated in the foregoing remarks. To present the facts under this view accurately and comprehensively, it would be necessary to give an account of the succession of appearances in almost every case distinctly; for, very few cases are to be found in which precisely the same appearances are presented, occurring in the same order of succession and time. But this minuteness of detail would not subserve any interesting or important purposes, since it is, obviously, impracticable to deduce any fixed laws regulating the succession of appearances, or to arrive at any practical results. It will suffice, therefore, to dismiss this branch of the subject with a few general remarks. The cases differed considerably as respects the number and kind of appearances which, respectively, they presented during the febrile career. In some cases the tongue remained simply furred; in others, it was furred at first, and thickly coated, subsequently; in others, it was furred, or thickly coated, early in the career, becoming more or less dry subsequently, and sometimes alternately moist and dry at different times. In a smaller proportion of cases the various other appearances occurred—in some cases one only of these appearances, and in others several—viz., tremulousness, redness, fissures, etc. All this, indeed, would be inferred from the account previously given of the various appearances under distinct heads. And it would be sufficient to say, under this division of the subject, that the analysis reveals nothing with reference to the number and succession of appearances in individual cases, which has any marked bearing on the distinctions between the two types of fever, or the relations of the tongue to other symptoms; moreover, the number and succession of appearances do not differ in any striking particulars in the cases which proved fatal, from some of those which ended in recovery.

In conclusion, with respect to the appearances of the tongue in Continued Fever, judging from the results of the present analysis, their value, as symptoms, cannot be considered very great. Here, as in the case of other symptoms, it does not enter into my plan to inquire to what extent similar appearances are to be observed in other diseases than Continued Fever. This, it is obvious, is a very important inquiry in order to determine in how far the phenomena described are peculiar to the disease under consideration. With regard to this comparison, the most accurate and extensive collection of recorded observations are doubtless to be found in the researches of Louis. But as regards the class of symptoms just considered, every practical reader is already aware that those which have been enumerated are not, in an eminent degree, distinctive of Continued Fever, although, probably several of them are oftener found in connection with that disease than in most other acute affections. As a general remark, however, they do not possess any very special significancy; in other words, they have not in the present state of knowledge very important bearings on the diagnosis, pathology, prognosis, or therapeutics. The results of enumeration and comparison in their application to this class of symptoms, are rather negative than positive. But here, as in other instances, by tending to disprove some errors which are more or less in vogue, these results are of scarcely less value, than if they disclosed important practical truths.

(*To be Continued.*)

